# *Notes from the Field:* E-cigarette Use Among Middle and High School Students — United States, 2022

**DOI:** 10.15585/mmwr.mm7140a3

**Published:** 2022-10-07

**Authors:** Maria Cooper, Eunice Park-Lee, Chunfeng Ren, Monica Cornelius, Ahmed Jamal, Karen A. Cullen

**Affiliations:** ^1^Center for Tobacco Products, Food and Drug Administration; ^2^Office on Smoking and Health, National Center for Chronic Disease Prevention and Health Promotion, CDC.

Since 2014, e-cigarettes have been the most commonly used tobacco product among U.S. middle and high school students (*1*). Most e-cigarettes contain nicotine, which is highly addictive, can harm the developing adolescent brain, and can increase risk for future addiction to other drugs (*2*). Among middle and high school current e-cigarette users (i.e., use on ≥1 day during the past 30 days), use of disposable e-cigarette devices* increased significantly between 2019 and 2020 (*3*) and was the most commonly used device type reported in 2021 (*4*). In 2020 and 2021, approximately eight in 10 middle and high school students who used e-cigarettes reported using flavored e-cigarettes (*4**,**5*). CDC and the Food and Drug Administration (FDA) analyzed nationally representative data from the 2022 National Youth Tobacco Survey (NYTS), a school-based, cross-sectional, self-administered survey conducted during January 18–May 31, 2022,^†^ using a web-based survey instrument and administered to U.S. middle school (grades 6–8) and high school (grades 9–12) students.^§^ Participating students could complete the survey whether they were physically in school or at home engaging in remote learning; 99.3% of students reported completing the survey in school. Current e-cigarette use was assessed overall and by frequency of use, device type, flavors, and brands used (any brand used and usual brand used).^¶^ Weighted prevalence estimates and population totals were calculated.** The NYTS study protocol was reviewed and approved by CDC’s institutional review board.^††^

In 2022, 14.1% of high school students and 3.3% of middle school students reported current e-cigarette use (Table). Among current e-cigarette users, 42.3% reported using e-cigarettes frequently,^§§^ including 46.0% of high school students and 20.8% of middle school students; daily use was reported among 27.6% of current e-cigarette users, including 30.1% of high school students and 11.7% of middle school students. Among current e-cigarette users, the types of devices most often used were disposables (high school = 57.2%; middle school = 45.8%), followed by prefilled or refillable pods or cartridges (high school = 25.7%; middle school = 21.6%), and tanks or mod systems (high school = 5.9%; middle school = 9.8%), with 11.2% of high school students and nearly 23% of middle school students reporting not knowing the type of e-cigarette device used.

**TABLE:** Prevalence of current (past 30-day) e-cigarette use,* overall and by selected characteristics and school level — National Youth Tobacco Survey, United States, 2022

Characteristic	Overall		High school		Middle school	
	Estimated weighted no.^†^	% (95% CI)	Estimated weighted no.^†^	% (95% CI)	Estimated weighted no.^†^	% (95% CI)
**Among all students (N = 28,291)**						
Current use of e-cigarettes	**2,550,000**	**9.4 (8.0–11.1)**	2,140,000	14.1 (12.4–16.0)	380,000	3.3 (2.6–4.2)
**Among current e-cigarette users**						
**Frequency of use during past 30 days**						
1–5 days	**1,030,000**	**40.6 (37.2–44.1)**	790,000	37.2 (33.4–41.1)	230,000	60.0 (53.3–66.3)
6–19 days	**430,000**	**17.1 (14.2–20.4)**	360,000	16.8 (13.9–20.2)	70,000	19.3 (12.7–28.3)
20–30 days	**1,080,000**	**42.3 (38.5–46.3)**	980,000	46.0 (41.6–50.4)	80,000	20.8 (15.8–26.8)
**Daily e-cigarette use** ^§^	**700,000**	**27.6 (24.5–31.0)**	640,000	30.1 (26.6–33.9)	40,000	11.7 (8.0–16.7)
**Device type most often used^¶^**						
Disposables	**1,390,000**	**55.3 (49.5–61.0)**	1,210,000	57.2 (51.7–62.6)	170,000	45.8 (34.5–57.6)
Prefilled or refillable pods or cartridges	**630,000**	**25.2 (19.7–31.5)**	540,000	25.7 (20.2–32.0)	80,000	21.6 (12.8–33.9)
Tanks or mod system	**160,000**	**6.7 (5.3–8.4)**	120,000	5.9 (4.5–7.8)	30,000	9.8 (7.1–13.5)
Don't know the type	**320,000**	**12.8 (10.2–16.1)**	230,000	11.2 (8.6–14.4)	80,000	22.8 (17.0–29.9)
Any brand**						
Puff Bar	**730,000**	**29.7 (25.5–34.4)**	610,000	29.3 (25.0–34.0)	110,000	30.9 (21.3–42.4)
Vuse	**580,000**	**23.6 (17.9–30.3)**	490,000	23.8 (17.9–30.9)	70,000	20.9 (13.2–31.3)
JUUL	**540,000**	**22.0 (17.8–26.9)**	440,000	21.2 (16.3–27.1)	80,000	23.8 (17.8–30.9)
SMOK (including NOVO)	**330,000**	**13.5 (10.8–16.6)**	290,000	14.3 (11.4–17.9)	20,000	7.8 (4.4–13.5)
NJOY	**200,000**	**8.3 (6.0–11.4)**	170,000	8.2 (5.6–11.7)	20,000	7.3 (4.3–12.1)
Hyde^††^	**180,000**	**7.3 (4.4–12.0)**	160,000	7.9 (4.6–13.3)	—^§§^	—^§§^
blu	**160,000**	**6.5 (4.9–8.6)**	110,000	5.6 (3.9–7.8)	30,000	10.2 (5.7–17.6)
STIG	**120,000**	**5.0 (3.6–6.8)**	90,000	4.7 (3.2–6.7)	—^§§^	—^§§^
Suorin	**110,000**	**4.8 (3.6–6.5)**	90,000	4.8 (3.5–6.5)	—^§§^	—^§§^
Logic	**100,000**	**4.3 (3.0–6.1)**	70,000	3.8 (2.5–5.6)	—^§§^	—^§§^
Mojo	**90,000**	**4.0 (2.8–5.5)**	70,000	3.7 (2.6–5.3)	—^§§^	—^§§^
Leap	**90,000**	**3.7 (2.6–5.2)**	60,000	3.0 (2.0–4.4)	—^§§^	—^§§^
Eonsmoke	**80,000**	**3.6 (2.4–5.3)**	60,000	2.9 (1.8–4.7)	—^§§^	—^§§^
Some other brand not listed	**790,000**	**32.2 (27.8–37.0)**	670,000	32.2 (27.4–37.4)	120,000	32.8 (25.5–41.0)
Not sure/Don’t know the brand	**700,000**	**28.3 (24.8–32.0)**	550,000	26.7 (22.7–31.1)	140,000	37.4 (29.7–45.8)
**Usual brand^¶¶^**						
Puff Bar	**350,000**	**14.5 (11.5–18.3)**	280,000	14.0 (10.9–17.9)	60,000	17.7 (11.0–27.2)
Vuse	**300,000**	**12.5 (8.3–18.3)**	260,000	13.1 (8.8–19.1)	—^§§^	—^§§^
Hyde^††^	**130,000**	**5.5 (3.1–9.6)**	—^§§^	—^§§^	—^§§^	—^§§^
SMOK (including NOVO)	**90,000**	**4.0 (2.8–5.8)**	80,000	4.4 (3.0–6.5)	—^§§^	—^§§^
JUUL	**—^§§^**	**—^§§^**	—^§§^	—^§§^	20,000	6.7 (3.8–11.5)
No usual brand	**80,000**	**3.3 (2.3–4.7)**	50,000	2.9 (1.9–4.4)	—^§§^	—^§§^
Some other brand not listed	**520,000**	**21.8 (17.7–26.6)**	450,000	22.6 (17.9–28.1)	60,000	17.5 (12.2–24.3)
Not sure/Don’t know the brand	**590,000**	**24.8 (21.2–28.8)**	470,000	23.6 (19.5–28.2)	110,000	31.9 (25.4–39.0)
Flavored e-cigarette use***						
Yes	**2,110,000**	**84.9 (82.4–87.2)**	1,790,000	85.5 (82.9–87.8)	300,000	81.5 (75.0–86.6)
No	**230,000**	**9.3 (7.7–11.2)**	190,000	9.3 (7.5–11.6)	30,000	9.5 (6.5–13.8)
Don't know	**140,000**	**5.7 (4.5–7.3)**	100,000	5.2 (4.1–6.5)	30,000	9.0 (5.7–13.9)
**Among current flavored e-cigarette users**						
**Flavor type used^†††^**						
Fruit	**1,450,000**	**69.1 (65.4–72.6)**	1,220,000	68.5 (64.4–72.3)	210,000	71.1 (63.9–77.3)
Candy, desserts, or other sweets	**800,000**	**38.3 (33.8–42.9)**	660,000	37.3 (32.6–42.2)	130,000	43.6 (36.3–51.3)
Mint	**610,000**	**29.4 (25.6–33.5)**	540,000	30.3 (25.9–35.1)	70,000	23.7 (18.9–29.3)
Menthol	**550,000**	**26.6 (21.0–33.1)**	500,000	28.2 (22.2–35.2)	40,000	16.2 (10.3–24.6)
Alcoholic drinks	**150,000**	**7.6 (5.6–10.2)**	120,000	6.8 (4.7–9.8)	30,000	10.8 (7.0–16.1)
Chocolate	**80,000**	**4.3 (3.1–5.9)**	60,000	3.8 (2.7–5.3)	—^§§^	—^§§^
Clove or spice	**60,000**	**2.9 (1.9–4.6)**	40,000	2.6 (1.6–4.2)	—^§§^	—^§§^
Some other flavor not listed	**240,000**	**11.7 (10.1–13.6)**	200,000	11.7 (9.9–13.7)	30,000	11.9 (8.0–17.5)

* Past 30-day use of e-cigarettes was determined by the question, “During the past 30 days, on how many days did you use e-cigarettes?” Current use was defined as use on ≥1 day during the past 30 days.

^†^ Estimated total number of users was rounded down to the nearest 10,000 students. Overall population totals might not directly sum to corresponding estimates by school level because of rounding or inclusion of students who did not self-report grade level.

^§^ Daily e-cigarette use was defined as use on all 30 of the past 30 days.

^¶^ Device type was determined by the question, “Which of the following best describes the type of e-cigarette you have used in the past 30 days? If you have used more than one type, please think about the one you use most often.”

** All current e-cigarette users were asked, “During the past 30 days, what e-cigarette brands did you use? (Select one or more).” Those who selected “some other brand(s) not listed here” could provide a write-in response. Write-in responses corresponding to an original response option were recoded. Data for Posh are not shown because of statistically unreliable estimates.

^††^ Hyde was not included in the list of prespecified response options, but it was the most commonly provided write-in response for “some other brand.” Write-in responses for Hyde were recoded, and all remaining responses were maintained as “some other brand.”

^§§^ Data were statistically unreliable because of unweighted denominator <50 or a relative SE >30%.

^¶¶^ If a single brand was selected for the question, “During the past 30 days, what e-cigarette brands did you use (Select one or more),” it was reported as their usual brand. Those who selected one or more brand were asked, “During the past 30 days, what brand of e-cigarettes did you usually use? (Choose only one answer).” Those who selected “some other brand(s) not listed here” could provide a write-in response. Write-in responses corresponding to an original response option were recoded. Data for blu, Eonsmoke, Leap, Logic, Mojo, NJOY, Posh, STIG, and Suorin are not shown because of statistically unreliable estimates.

*** Flavored e-cigarette use was assessed by response to the question, “Were any of the e-cigarettes that you used in the past 30 days flavored to taste like menthol, mint, clove or spice, alcoholic drinks, candy, fruit, chocolate, or any other flavor?”

^†††^ Flavor type was determined by response to the question, “What flavors were the e-cigarettes that you have used in the past 30 days? (Select one or more).” Those who selected “some other flavor not listed here” could provide a write-in response. Write-in responses corresponding to an original response option were recoded.

Among current e-cigarette users, Puff Bar was the most commonly reported brand used in the past 30 days by both middle and high school students (29.7%), followed by Vuse (23.6%), JUUL (22.0%), SMOK (13.5%), NJOY (8.3%), Hyde (7.3%), and blu (6.5%). Among current e-cigarette users, 14.5% reported that the brand they usually used was Puff Bar, followed by Vuse (12.5%), Hyde (5.5%), and SMOK (4.0%). Approximately one fifth (21.8%) of current e-cigarette users reported “some other brand” as their usual brand.

Among current e-cigarette users overall, 84.9% used flavored e-cigarettes; of these, the reported flavor types, in descending order of use, were fruit (69.1%); candy, desserts, or other sweets (38.3%); mint (29.4%); and menthol (26.6%). A similar pattern was observed among current users of flavored disposable e-cigarettes: fruit (75.2%); candy, desserts, or other sweets (40.4%); mint (29.6%); and menthol (16.7%) (Supplementary Table, https://stacks.cdc.gov/view/cdc/121630). Among current users of flavored pods or cartridges, the reported flavor types used were fruit (58.4%); menthol (53.9%); candy, desserts, or other sweets (30.3%); and mint (27.6%). Among current users of flavored tanks or mod systems, the reported flavor types used were fruit (69.6%); candy, desserts, or other sweets (47.7%); mint (40.1%); and menthol (35.2%).

In 2022, 2.55 million U.S. middle and high school students currently used e-cigarettes. Most reported using flavored products, and, among those students, approximately seven of 10 used fruit flavors. Disposable products were the most commonly reported device type. Further, among middle and high school students who used e-cigarettes, approximately four in 10 reported frequent use, and approximately one in four reported daily use. The use of tobacco products in any form, including e-cigarettes, by middle and high school students is unsafe. Sustained implementation of comprehensive tobacco prevention and control strategies at the national, state, and local levels,^¶¶^ coupled with FDA regulation and enforcement, is critical to addressing e-cigarette use among middle and high school students (*2*).

## Footnotes

* Disposable e-cigarettes come prefilled with e-liquid, and the entire device is designed to be discarded after a single use. Other devices have pods or cartridges that hold the e-liquid. Some pods or cartridges come prefilled with e-liquid and are replaced after use, and others can be refilled by the user. Tank or mod-type devices can also be refilled, but are also usually customizable, allowing the user to change the temperature or voltage, nicotine concentrations, and add accessories.

† In 2022, 28,291 students from 341 schools participated (overall response rate = 45.2%).

§ Because of changes in methodology, including differences in survey administration and data collection procedures, the ability to compare estimates from 2022 with those from previous NYTS waves is limited; differences between estimates might be due to changes in methodology, actual behavior, or both. The NYTS was conducted in schools using an electronic tablet in 2019 and 2020. Because of COVID-19 concerns, the 2021 NYTS was conducted using web-based data collection, with approximately one half (50.8%) of students completing it in school. The 2022 NYTS was also conducted using web-based data collection; however, nearly all (99.3%) students completed the survey in school.

¶ Brand response options were as follows: blu, Eonsmoke, JUUL, Leap, Logic, Mojo, NJOY, Posh, Puff Bar, SMOK (including NOVO), STIG, Suorin, Vuse, “some other brand(s) not listed here,” and “I don’t know the brand.” Those who selected “some other brand(s) not listed here” could provide a write-in response. Write-in responses were recoded into valid responses. One additional brand, Hyde, is reported based on the write-in responses. As a result, estimates of Hyde use might be underestimated.

** Weighted population estimates were rounded down to the nearest 10,000 students.

†† 45 C.F.R. part 46; 21 C.F.R. part 56.

§§ Frequent e-cigarette use was defined as use on ≥20 days in the past 30 days. Daily e-cigarette use was defined as use on all of the past 30 days. These estimates are not mutually exclusive.

¶¶ CDC’s website has resources and information related to tobacco prevention and control at the local, state, and national levels, including information to guide parents, teachers, and school administrators and coaches in an informed discussion on e-cigarettes with young persons. https://www.cdc.gov/tobacco/index.htm
